# Trends in the Treatment of Adolescent Clavicle Fractures: Are We Listening to the Evidence?

**DOI:** 10.5435/JAAOSGlobal-D-22-00277

**Published:** 2023-02-03

**Authors:** Matthew W. Cole, Lacee K. Collins, McCayn M. Familia, Timothy J. Skalak, Olivia C. Lee, William F. Sherman

**Affiliations:** From the Department of Orthopaedic Surgery, Tulane University School of Medicine, New Orleans, LA, and Department of Orthopaedic Surgery, LSUHSC School of Medicine, New Orleans, LA.

## Abstract

From 1999 to 2011, studies demonstrated an increasing trend toward surgical management of adolescent clavicle fractures. The purpose of this study was to examine more recent trends of surgical management of closed clavicle fractures in adolescent patients over the past decade.

**Methods:**

A retrospective cohort study was conducted using the PearlDiver database. Patients with clavicle fractures from 2011 to 2021 were identified and stratified by age, sex, and year of their fracture. Categorical variables were compared with a chi square test, and continuous variables were compared with the Welch *t* test or Mann-Whitney *U* test.

**Results:**

Overall, there was a significant increase in the percentage of patients surgically treated by open reduction and internal fixation from 2016 to 2021 compared with 2011 to 2015 (8.58% vs. 7.34%, *P* < 0.001). When stratified by age, both the 10 to 14-year group (3.80% vs. 3.10%, *P* < 0.001) and the 15 to 18-year group (15.41% vs. 12.84%, *P* < 0.001) demonstrated significant increases in the percentage of patients surgically treated.

**Conclusion:**

Despite increasing literature demonstrating high revision surgery rates for surgical treatment of adolescent clavicle fractures with no difference in functional outcomes, this study demonstrated a notable increase in the rate of surgical treatment of adolescent clavicle fractures from 2011 to 2021 in the United States.

Clavicle fractures are among the most common fractures in adolescent patients, encompassing 7% to 15% of fractures in this population.^1^ These fractures are often caused by trauma or direct falls onto the lateral shoulder and have been found to respond well to nonsurgical treatment.^2^ An epidemiological study on the Function after Adolescent Clavicle Trauma and Surgery cohort by Ellis et al^3^ demonstrated that these fractures are frequently sports-related (66%) and occur in male patients at a higher rate than female patients (3.8:1).

Studies have demonstrated an increasing trend toward surgical management of adolescent clavicle fractures from 1999 to 2011.^4-6^ Factors which have been demonstrated to be associated with a higher rate of surgical treatment include male sex and private insurance coverage.^6-8^ By contrast, studies over the past decade continue to demonstrate equal or better outcomes for adolescent patients with closed clavicle fractures who are treated nonsurgically, even in those with markedly displaced or translated fracture patterns.^9,10,11,12,13,14,15^ In addition, surgical fixation of adolescent clavicle fractures is also associated with a very high overall complication rate and rate of revision surgery for hardware removal.^16,17^ Although most patients can be successfully treated nonsurgically, surgical treatment is indicated in cases of soft-tissue compromise, notable displacement, open fracture, or the presence of multiple injuries.^18,19^

As the literature demonstrating no notable benefit of surgical treatment has continued to accrue, a more current analysis of trends of open reduction and internal fixation (ORIF) versus nonsurgical treatment is warranted to examine whether surgeons are following the newest available evidence. The purpose of this study was to examine trends of surgical versus nonsurgical management of adolescent clavicle fractures over the past decade. With the number of recent studies highlighting no difference in outcomes with ORIF, it was hypothesized that there would be a decreasing trend in surgical fixation of these fractures. A secondary analysis was conducted to evaluate whether sex or insurance status had an influence on treatment choices.

## Methods

### Data Source and Study Design

Patient records were queried from the PearlDiver Mariner Database (PearlDiver Inc), a commercially available administrative claims database, which contains deidentified patient data from the inpatient and outpatient settings. The database contains the medical records of patients across the United States from 2010 to Q1 of 2021, which are collected by an independent data aggregator. This study used the “M151Ortho” data set within PearlDiver, which contains a random sample of 151 million patients. All health insurance payors are represented including commercial, private, and government plans. Researchers extract data using Current Procedural Terminology (CPT) and International Classification of Diseases, Ninth and 10th revision (ICD-9/ICD-10) codes. Institutional review board exemption was granted because provided data were deidentified and compliant with the Health Insurance Portability and Accountability Act. No outside funding was received for this study.

A retrospective cohort study was conducted to investigate the trends of surgical treatment of adolescent clavicle fractures for patients aged 10 to 18 years. Patients with clavicle fractures were identified using relevant ICD-9/10 diagnosis codes. Patients with open clavicle fractures were excluded. Patients who underwent surgical treatment by ORIF were identified by the presence of CPT-23515 on the same day or within 1 month after the diagnosis code for clavicle fracture. Patients who underwent nonsurgical treatment were identified by the absence of CPT-23515 on the same day or after the diagnosis code for clavicle fracture. Patients were stratified by sex and into two age cohorts of 10 to 14 years and 15 to 18 years. These cohorts were further subdivided by year of their fracture to identify trends in treatment (Appendix A, http://links.lww.com/JG9/A255).

### Complications

Complications after surgical fixation within 2 years were identified using relevant CPT and ICD-9/10 codes. Revision ORIF was defined using CPT-20680 and CPT-23515 on the same day. Complications queried were hardware removal (IPR), revision ORIF, incision and débridement (I&D), and scar revision. Only patients from 2011 to 2018 were included in the query of complication rates to ensure there was a full 2 years of follow-up data. The rate of conversion from nonsurgical treatment to ORIF was also queried using the presence of CPT-23515 after 1 month of initial nonsurgical treatment. The complication rates were also stratified by sex and into two age cohorts of 10 to 14 years and 15 to 18 years (Appendix B, http://links.lww.com/JG9/A255).

### Statistical Analysis

Statistical analyses were conducted using R statistical software (Version 4.1.0; R Project for Statistical Computing) integrated within the PearlDiver software with an α level set at 0.05. Categorical variables were compared with a chi square test, and continuous variables were compared with the Welch *t* test or Mann-Whitney *U* test.

## Results

### Study Population and Trends in Treatment

A total of 737,541 patients with closed clavicle fractures were identified, including 68,084 patients (9.2%) aged 10 to 14 years and 51,080 patients (6.9%) aged 15 to 18 years. After filtering by year of injury, 61,290 patients aged 10 to 14 years and 45,095 patients aged 15 to 18 years had their injury occur from the beginning of Q1 of 2011 through the end of Q1 of 2021. After filtering all included patients by sex, 23,873 (22.4%) were female and 82,482 (77.6%) were male. Among all included patients, 82,522 (77.6%) had commercial insurance and 20,028 (18.8%) had Medicaid.

### Surgical Treatment Rates by Age

**Figure 1:**
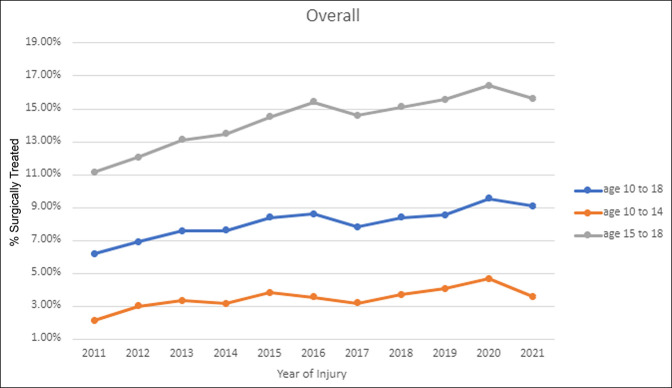
Graph showing trends in treatment of adolescent clavicle fractures overall for 10 to 18 years and by age groups 10 to 14 years and 15 to 18 years.

Overall, there was a significant increase in the percentage of patients surgically treated by ORIF from 2016 to 2021 compared with 2011 to 2015 (8.58% vs. 7.34%, *P* < 0.001). When stratified by age, both the 10 to 14-year group (3.80% vs. 3.10%, *P* < 0.001) and the 15 to 18-year group (15.41% vs. 12.84%, *P* < 0.001) demonstrated significant increases in the percentage of patients surgically treated from 2016 to 2021 compared with 2011 to 2015 (Figure 1). Overall, only 104 patients (0.11%) who were treated nonsurgically for the first month were subsequently converted to ORIF.

### Surgical Treatment Rates by Sex

**Figure 2:**
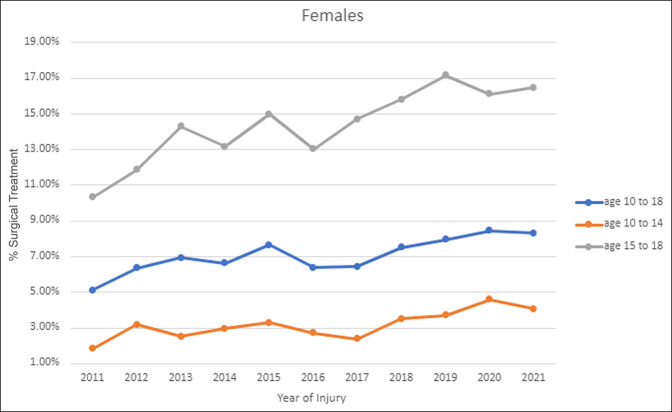
Graph showing trends in treatment of female adolescent clavicle fractures overall for 10 to 18 years and by age groups 10 to 14 years and 15 to 18 years.

**Figure 3:**
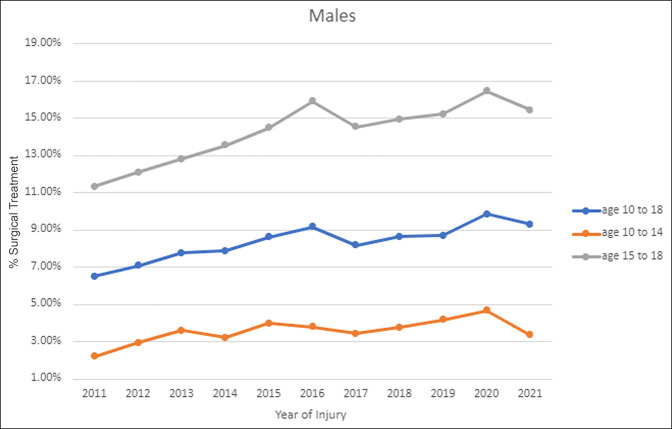
Graph showing trends in treatment of male adolescent clavicle fractures overall for 10 to 18 years and by age groups 10 to 14 years and 15 to 18 years.

When stratified by sex, both female (7.38% vs. 6.54%, *P* = 0.012) and male (8.93% vs. 7.58%, *P* < 0.001) patients demonstrated significant increases in the percentage of patients surgically treated from 2016 to 2021 compared with 2011 to 2015. Female patients in the 10 to 14-year group (3.40% vs. 2.76%, *P* = 0.024) and 15 to 18-year group (15.36% vs. 12.91%, *P* = 0.001) demonstrated significant increases in the percentage of patients surgically treated from 2016 to 2021 compared with 2011 to 2015 (Figure 2). Male patients in the 10 to 14-year group (3.93% vs. 3.21%, *P* < 0.001) and 15 to 18-year group (15.43% vs. 12.85%, *P* < 0.001) demonstrated significant increases in the percentage of patients surgically treated from 2016 to 2021 compared with 2011 to 2015 (Figure 3). Male patients were significantly more likely than female patients to receive surgical treatment overall from 2011 to 2021 (8.25% vs. 6.95%, *P* < 0.001) and for both 2011 to 2015 (7.58% vs. 6.54%, *P* < 0.001) and 2016 to 2021 (8.93% vs. 7.38%, *P* < 0.001). However, when stratified by age, there was no difference between male and female patients aged 15 to 18 years overall from 2011 to 2021 (14.09% vs. 14.02%, *P* = 0.877) or for 2011 to 2015 (12.85% vs. 12.91%, *P* = 0.951) and 2016 to 2021 (15.43% vs. 15.36%, *P* = 0.920). Male patients aged 10 to 14 years were significantly more likely than female patients to receive surgical treatment overall from 2011 to 2021 (3.58% vs. 3.08%, *P* = 0.004) and for 2011 to 2015 (3.21% vs. 2.76%, *P* = 0.043) and 2016 to 2021 (3.93% vs. 3.40%, *P* = 0.040).

### Surgical Treatment Rates by Insurance Plan

When stratified by insurance type, patients with commercial insurance were significantly more likely to receive surgical treatment overall from 2011 to 2021 (9.15% vs. 7.94%, *P* < 0.001) and for both 2011 to 2015 (8.46% vs. 7.37%, *P* < 0.001) and 2016 to 2021 (9.90% vs. 8.48%, *P* < 0.001). Both Medicaid (8.48% vs. 7.37%, *P* = 0.003) and commercial insurance (9.90% vs. 8.46%, *P* < 0.001) patients demonstrated significant increases in the percentage of patients surgically treated from 2016 to 2021 compared with 2011 to 2015.

### Overall Complication Rates

Among patients who were treated surgically, there was a significant decrease in overall revision surgeries (IPR, I&D, revision ORIF, and scar revision) from 2015 to 2018 compared with 2011 to 2014 (16.79% vs. 21.82%, *P* < 0.001). A significant decrease was observed in IPR from 2015 to 2018 compared with 2011 to 2014 (15.93% vs. 20.50%, *P* < 0.001). A significant decrease was also observed in scar revision from 2015 to 2018 compared with 2011 to 2014 (1.39% vs. 2.07%, *P* = 0.046). However, rates of both I&D (0.44% vs. 0.36%, *P* = 0.711) and revision ORIF (0.74% vs. 0.71%, *P* = 1) were not significantly different from 2015 to 2018 compared with 2011 to 2014.

### Complication Rates by Sex

Among female patients who were treated surgically, there was no significant difference in the total rate of revision surgery (IPR, I&D, revision ORIF, and scar revision) (22.31% vs. 25.92%, *P* = 0.154) or IPR (21.03% vs. 24.32%, *P* = 0.186), I&D (*P* = 0.154), revision ORIF (*P* = 0.154), or scar revision (2.41% vs. 2.40%, *P* = 1) alone from 2015 to 2018 compared with 2011 to 2014. To protect patient identities, the database software does not report actual patient counts when defined cohorts contain less than 10 patients. As a result, the percentages of both I&D and revision ORIF are unable to be reported because of patient counts less than 11.

Among male patients who were treated surgically, there was a significant decrease in the total rate of revision surgeries (IPR, I&D, revision ORIF, and scar revision) from 2015 to 2018 compared with 2011 to 2014 (15.54% vs. 20.78%, *P* < 0.001). A significant decrease was observed in IPR from 2015 to 2018 compared with 2011 to 2014 (14.78% vs. 19.53%, *P* < 0.001). A significant decrease was also noted in scar revision from 2015 to 2018 compared with 2011 to 2014 (1.16% vs. 1.98%, *P* = 0.022). However, rates of both I&D (0.47% vs. 0.32%, *P* = 0.258) and revision ORIF (0.65% vs. 0.77%, *P* = 0.742) were not significantly different from 2015 to 2018 compared with 2011 to 2014.

## Discussion

This study demonstrated a notable increase in the rate of surgical treatment of adolescent clavicle fractures from 2011 to 2021 in the United States. This trend toward more surgical treatment was observed in all cohorts, regardless of age or sex. However, male patients and older children (15 to 18 years) had a more notable increase in surgical treatment compared with female patients and younger children (10 to 14 years). This aligns with previous literature on the trend of treatment of these fractures. A single-institution study by Suppan et al^5^ demonstrated an increase in the rate of surgical treatment of midshaft clavicle fractures in adolescents from 1999 to 2011. Yang et al demonstrated that older adolescents (15 to 19 years) had the largest increase in surgical treatment from 2007 to 2011.^6^ A single-institution study in Finland demonstrated an increase in the surgical treatment rate since 2008.^20^ The increased rate of surgical treatment demonstrated from 1999 to 2011 has continued into 2021.

This observed trend is not supported by published literature on outcomes of surgical versus nonsurgical treatment of closed adolescent clavicle fractures. Although fracture characteristics were not obtainable using the database, several studies have demonstrated no difference in outcomes, regardless of fracture type, amount of shortening, and even malunion. Parry et al^10^ demonstrated that there was no difference in range of motion, isometric strength, or abduction fatigue between surgical and nonsurgical treatments of shortened midshaft fractures. A study on the Function after Adolescent Clavicle Trauma and Surgery cohort by Hayworth et al^14^ demonstrated no benefit to surgical treatment of completely displaced clavicle shaft fractures in adolescents in patient-reported quality of life, satisfaction, or shoulder function. Bae et al^21^ demonstrated no meaningful loss of motion or abduction/adduction strength even with an established diagnosis of malunion. A study by Schulz et al^9^ demonstrated that for displaced, shortened, midshaft clavicle fractures, nonsurgical treatment resulted in no differences between the injured and uninjured limbs for pain or subjective function, regardless of the fracture type, amount of clavicle shortening, patient age, or sports participation. A retrospective study by Hagstrom et al^22^ demonstrated equivalent outcomes for the surgical and nonsurgical groups at intermediate and long-term follow-ups. At long-term follow-up, Ng et al^12^ demonstrated excellent results for nonsurgical treatment and concluded that the relative indications for surgery in adults do not seem to apply in adolescents. A study by Calder et al^23^ even demonstrated that there is no need for radiographic follow-up with isolated, uncomplicated clavicle fractures. In one conflicting study, a meta-analysis by Gao et al^24^ demonstrated faster return to activity and superior Constant scores, but did note a higher complication rate for surgical management, which frequently required secondary operation. Ames et al^25^ demonstrated a 22% refracture rate in those treated nonsurgically, but also that 27% of those treated surgically required a second operation. However, a meta-analysis by Nawar et al^26^ demonstrated no difference in return to sports between surgical and nonsurgical treatments. This is similar to a study by Riiser et al., which demonstrated good outcomes in long term with plate fixation, intramedullary nailing, and nonsurgical treatments.^27^

Surgical treatment is not without risk of complications or the need for revision surgery. In this study, there was a notable revision surgery rate observed for the surgical cohort within 2 years of initial surgery. This result aligns with prior literature on complication rates after surgical treatment of adolescent clavicle fractures. A retrospective cohort study of 36 patients by Li et al^28^ demonstrated a complication rate of 86%, with implant prominence or irritation being the most common and 41.7% of these patients going on to subsequent hardware removal. A study by Carrillo et al^29^ demonstrated a revision surgery rate of 15.9% most frequently for removal of the implant (92.5%). A retrospective cohort study by Luo et al^30^ demonstrated a complication rate of 21.7%. Although some surgeons plan for hardware removal and thus may not consider it a complication, an additional surgical event is not without risk and cost.^31^ Regardless, a high complication and revision surgery rate, especially for IPR, is consistently noted throughout the literature. It should be noted that in this study, the rate of IPR decreased in 2016 to 2021 compared with 2011 to 2015. One possible explanation for this could be that surgeons are increasing the use of precontoured plates, which have been demonstrated to reduce the rate of IPR.^32,33^ Other possibilities include changes in placement of the plate as studies have demonstrated that anterior-inferior plating results in low rates of IPR.^34^

In the adult population, surgical management of clavicle fractures has a larger role and can provide better function and less disability following some fracture patterns, specifically in fractures that are markedly displaced or comminuted.^35^ A systematic review by Zlowodzki et al^36^ demonstrated fracture displacement, comminution, number of fragments, and older age to be associated with long-term sequelae after nonsurgical treatment. Some indications for adult treatment include z-type fractures, greater than 2 cm of shortening, greater than 100% displacement, floating shoulder, polytrauma, neurovascular injury, skin tenting, or open fractures.^37-39^ Apart from open fractures, such indications for surgical fixation do not apply to adolescent clavicle fractures.^13^ A 2011 survey of 302 pediatric orthopaedic surgeons demonstrated that 48.48% of respondents were more inclined to choose surgical treatment because of recent adult literature.^40^ A study by Luo et al^30^ demonstrated that pediatric fellowship-trained orthopaedic surgeons were less likely to treat pediatric fractures surgically than nonpediatric orthopaedic surgeons (10.3% vs. 32.6%).

Application of adult literature to the pediatric/adolescent population is likely inappropriate because of the timing of clavicle ossification. In contrast to long bones such as the tibia where ossification centers close between 15 and 16 years for female patients and 17 and 18 years for male patients, the medial epiphysis of the clavicle does not completely ossify until 20 years, with the ossification centers often remaining unfused until 25 years.^18,41^ Bone healing potential is greater in the pediatric/adolescent population versus in adults. Although the three basic phases of bone healing occur in both populations, a pediatric bone that is still growing is already in an osteogenic phase while adult bones must return to this phase, leading to slower healing times in adult versus pediatric patients.^42^ In addition, pediatric bones that are still growing can more easily correct defects of fracture alignment or angulation, leaving little, if any, signs of a previous fracture.^42^ In this study, older children had surgical treatment at a much higher rate, suggesting both pediatric and adult orthopaedic surgeons may be leaning toward adult treatment when taking care of older children.

In this study, male patients were overall markedly more likely to receive surgical treatment than female patients from 2011 to 2021. However, when stratified by age, male and female patients aged 15 to 18 years demonstrated no difference in the percentage receiving surgical treatment over any of the study periods. By contrast, male patients aged 10 to 14 years were markedly more likely to receive surgical treatment at all study periods than female patients. A study by Yang et al^6^ on adolescent clavicle fractures demonstrated a trend toward more male patients being managed with ORIF, but their data did not reach significance and the study did not analyze differences in age groups between male and female patients.

Adolescent patients with private insurance were markedly more likely to receive surgical treatment than those with Medicaid. These results align with prior literature demonstrating that adult patients with clavicle fractures were more likely to receive surgical treatment if they had private insurance compared with the uninsured or those with Medicare or Medicaid.^7,8^ These data suggest that, similar to the literature on adults with Medicaid, children with Medicaid are not being treated the same as those with private insurance. The cause of these differences requires more exploration. A study by Lindsay et al^43^ demonstrated that patients with Medicare or Medicaid were more passive in the shared decision-making process between surgeon and patient while younger patients and those who achieved higher education levels desired more decision-making responsibility. This difference in shared decision making is a possible contributing factor, but the cause is likely multifactorial. Other possible contributing factors include differences in reimbursement between the insurance plans and access-to-care disparities. A study by Iobst et al. randomly selected five orthopaedic offices per state and demonstrated that only 59 of 250 (23.6%) would see a pediatric fracture patient with Medicaid.^44^ The same study demonstrated that offices in the 10 states with the lowest Medicaid reimbursement only offered an appointment 6% of the time while offices in the 10 states with the highest Medicaid reimbursement offered appointments 44% of the time.^44^ In addition, because there is a high revision surgery and complication rate associated with surgical treatment of clavicle fractures, the additional cost associated with complications of surgical treatment could also be contributing to this disparity.

## Limitations

There are several limitations to this study. First, the possibility of coding errors is inherent with any analysis of administrative claims data. However, such instances are rare and made up only 0.7% of Medicare and Medicaid payments in 2021 and thus would have minimal effect on the outcomes demonstrated.^45^ Fracture characteristics such as location, fracture type, displacement, and angulation are not available in the database. In addition, given this study evaluated trends of clavicle fracture management and rates of surgical versus nonsurgical management using retrospective data, outcome information was not obtained. The database only contains United States data and, therefore, may not reflect global patterns in clavicle fracture treatment. Data on the surgeon fixing the fracture are not available in PearlDiver; thus, we are unable to obtain whether there is a difference in the rate of surgical treatment between pediatric fellowship-trained orthopaedic surgeons compared with nonpediatric fellowship-trained. This study defined nonsurgical treatment as no operation within 1 month of fracture diagnosis. Due to this cutoff, the nonsurgical cohort may be overestimated, therefore underestimating the surgical cohort. For example, if a patient initially presented to a surgeon 5 weeks after diagnosis and was indicated for surgical fixation at that visit, they would be analyzed as a nonsurgical patient with subsequent surgery. There is also the possibility of an influence in these trends of treatment owing to parental preference, but this is not able to be evaluated through a database study.

## Conclusion

From 2011 to 2021, there was a notable increase in the percentage of adolescent clavicle fractures being treated surgically, regardless of age or sex of the patient. Male patients and patients aged 15 to 18 years experienced a larger increase in surgical fixation relative to female patients and patients aged 10 to 14 years. Patients with private insurance were markedly more likely to receive surgical treatment than those with Medicaid. Surgical treatment was associated with a high complication rate mostly because of the high revision surgery rate for hardware removal. This trend toward more surgical treatment is in direct opposition to the literature published over the past decade demonstrating outcomes of surgical treatment to be equivalent to nonsurgical treatment, regardless of fracture characteristics.

## References

[1] Hughes K Kimpton J Wei R : Clavicle fracture nonunion in the paediatric population: A systematic review of the literature. J Child Orthop 2018;12:2-8. 29456747 10.1302/1863-2548.12.170155 PMC5813118

[2] Bentley TP Hosseinzadeh S: Clavicle Fractures. Tampa, FL, StatPearls, 2022. 29939669

[3] Ellis HB Li Y Bae DS , FACTS Study Group: Descriptive epidemiology of adolescent clavicle fractures: Results from the FACTS (function after adolescent clavicle trauma and surgery) prospective, multicenter cohort study. Orthop J Sports Med 2020;8:232596712092134. 10.1177/2325967120921344 PMC7263158 32528990

[4] Pandya NK: Adolescent clavicle fractures: Is there a role for open reduction and internal fixation. Curr Rev Musculoskelet Med 2019;12:228-232. 30924049 10.1007/s12178-019-09553-7 PMC6542967

[5] Suppan CA Bae DS Donohue KS Miller PE Kocher MS Heyworth BE: Trends in the volume of operative treatment of midshaft clavicle fractures in children and adolescents: A retrospective, 12-year, single-institution analysis. J Pediatr Orthop B 2016;25:305-309. 26990058 10.1097/BPB.0000000000000301

[6] Yang S Werner BC Gwathmey FW: Treatment trends in adolescent clavicle fractures. J Pediatr Orthop 2015;35:229-233. 24992356 10.1097/BPO.0000000000000258

[7] Bliss RL Mora AM Krause PC: Does insurance status affect the management of acute clavicle fractures?. J Orthop Trauma 2016;30:269-272. 26618664 10.1097/BOT.0000000000000498

[8] Congiusta DV Amer KM Merchant AM Vosbikian MM Ahmed IH: Is insurance status associated with the likelihood of operative treatment of clavicle fractures?. Clin Orthop Relat Res 2019;477:2620-2628. 31764322 10.1097/CORR.0000000000000836 PMC6907309

[9] Schulz J Moor M Roocroft J Bastrom TP Pennock AT: Functional and radiographic outcomes of nonoperative treatment of displaced adolescent clavicle fractures. J Bone Joint Surg Am 2013;95:1159-1165. 23824383 10.2106/JBJS.L.01390

[10] Parry JA Van Straaten M Luo TD : Is there a deficit after nonoperative versus operative treatment of shortened midshaft clavicular fractures in adolescents?. J Pediatr Orthop 2017;37:227-233. 26327404 10.1097/BPO.0000000000000627

[11] O'Neill BJ Molloy AP Curtin W: Conservative management of paediatric clavicle fractures. Int J Pediatr 2011;2011:172571-172574. 22187568 10.1155/2011/172571 PMC3236468

[12] Ng N Nicholson JA Chen P Yapp LZ Gaston MS Robinson CM: Adolescent mid-shaft clavicular fracture displacement does not predict nonunion or inferior functional outcome at long-term follow-up. Bone Joint J 2021;103-B:951-957. 33934646 10.1302/0301-620X.103B5.BJJ-2020-1929.R1

[13] Swarup I Maheshwer B Orr S Kehoe C Zhang Y Dodwell E: Intermediate-term outcomes following operative and nonoperative management of midshaft clavicle fractures in children and adolescents: Internal fixation may improve outcomes. JB JS Open Access 2021;6:e20.00036. 10.2106/JBJS.OA.20.00036 PMC7963509 33748645

[14] Heyworth BE Pennock AT Li GY : Two-year functional outcomes of operative vs. Non-operative treatment of completely displaced midshaft clavicle fractures in adolescents: Results from A prospective, multicenter, level 2 study. Orthop J Sports Med 2019;7:2325967119S0042. 10.1177/03635465221114420 35984091

[15] Randsborg P-H Fuglesang HFS Røtterud JH Hammer O-L Sivertsen EA: Long-term patient-reported outcome after fractures of the clavicle in patients aged 10 to 18 years. J Pediatr Orthop 2014;34:393-399. 23965911 10.1097/BPO.0000000000000082

[16] Namdari S Ganley TJ Baldwin K : Fixation of displaced midshaft clavicle fractures in skeletally immature patients. J Pediatr Orthop 2011;31:507-511. 21654457 10.1097/BPO.0b013e318220ba48

[17] Mehlman CT Yihua G Bochang C Zhigang W: Operative treatment of completely displaced clavicle shaft fractures in children. J Pediatr Orthop 2009;29:851-855. 19934697 10.1097/BPO.0b013e3181c29c9c

[18] Paladini P Pellegrini A Merolla G Campi F Porcellini G: Treatment of clavicle fractures. Transl Med Unisa 2012;2:47-58. 23905044 PMC3728778

[19] Kubiak R Slongo T: Operative treatment of clavicle fractures in children: A review of 21 years. J Pediatr Orthop 2002;22:736-739. 12409898

[20] Sassi E Hannonen J Serlo W Sinikumpu J-J: Increase in surgical fixation of pediatric midshaft clavicle fractures since 2008. BMC Musculoskelet Disord 2022;23:173. 35197020 10.1186/s12891-021-04918-x PMC8864931

[21] Bae DS Shah AS Kalish LA Kwon JY Waters PM: Shoulder motion, strength, and functional outcomes in children with established malunion of the clavicle. J Pediatr Orthop 2013;33:544-550. 23752154 10.1097/BPO.0b013e3182857d9e

[22] Hagstrom LS Ferrick M Galpin R: Outcomes of operative versus nonoperative treatment of displaced pediatric clavicle fractures. Orthopedics 2015;38:e135-e138. 25665119 10.3928/01477447-20150204-62

[23] Calder JDF Solan M Gidwani S Allen S Ricketts DM: Management of paediatric clavicle fractures--is follow-up necessary? An audit of 346 cases. Ann R Coll Surg 2002;84:331-333. 10.1308/003588402760452457 PMC2504174 12398126

[24] Gao B Dwivedi S Patel SA Nwizu C Cruz AI: Operative versus nonoperative management of displaced midshaft clavicle fractures in pediatric and adolescent patients: A systematic review and meta-analysis. J Orthop Trauma 2019;33:e439-e446. 31633645 10.1097/BOT.0000000000001580

[25] Ames TD Mehlman CT Toy R Parikh SN: Comparative effectiveness of nonoperative versus operative treatment of completely displaced clavicle shaft fractures among children. Orthopedics 2022;45:373-377. 35947459 10.3928/01477447-20220805-03

[26] Nawar K Eliya Y Burrow S Peterson D Ayeni O de Sa D: Operative versus non-operative management of mid-diaphyseal clavicle fractures in the skeletally immature population: A systematic review and meta-analysis. Curr Rev Musculoskelet Med 2020;13:38-49. 31970646 10.1007/s12178-020-09604-4 PMC7083995

[27] Riiser MO Molund M: Long-term functional outcomes and complications in operative versus nonoperative treatment for displaced midshaft clavicle fractures in adolescents: A retrospective comparative study. J Pediatr Orthop 2021;41:279-283. 33606445 10.1097/BPO.0000000000001768

[28] Li Y Helvie P Farley FA Abbott MD Caird MS: Complications after plate fixation of displaced pediatric midshaft clavicle fractures. J Pediatr Orthop 2018;38:350-353. 27379787 10.1097/BPO.0000000000000832

[29] Carrillo LA Wu H-H Chopra A Callahan M Katyal T Swarup I: Rates of readmission and reoperation after operative management of midshaft clavicle fractures in adolescents. World J Orthop 2021;12:1001-1007. 35036342 10.5312/wjo.v12.i12.1001 PMC8696603

[30] Luo TD Ashraf A Larson AN Stans AA Shaughnessy WJ McIntosh AL: Complications in the treatment of adolescent clavicle fractures. Orthopedics 2015;38:e287-e291. 25901621 10.3928/01477447-20150402-56 PMC4899812

[31] Walton B Meijer K Melancon K Hartman M: A cost analysis of internal fixation versus nonoperative treatment in adult midshaft clavicle fractures using multiple randomized controlled trials. J Orthop Trauma 2015;29:173-180. 25233160 10.1097/BOT.0000000000000225

[32] VanBeek C Boselli KJ Cadet ER Ahmad CS Levine WN: Precontoured plating of clavicle fractures: Decreased hardware-related complications?. Clin Orthop Relat Res 2011;469:3337-3343. 21416203 10.1007/s11999-011-1868-0 PMC3210289

[33] Rongguang A Zhen J Jianhua Z Jifei S Xinhua J Baoqing Y: Surgical treatment of displaced midshaft clavicle fractures: Precontoured plates versus noncontoured plates. J Hand Surg 2016;41:e263-e266. 10.1016/j.jhsa.2016.06.007 27497801

[34] Baltes TPA Donders JCE Kloen P: What is the hardware removal rate after anteroinferior plating of the clavicle? A retrospective cohort study. J Shoulder Elbow Surg 2017;26:1838-1843. 28478898 10.1016/j.jse.2017.03.011

[35] Virtanen KJ Malmivaara AOV Remes VM Paavola MP: Operative and nonoperative treatment of clavicle fractures in adults. Acta Orthop 2012;83:65-73. 22248169 10.3109/17453674.2011.652884 PMC3278660

[36] Zlowodzki M Zelle BA Cole PA Jeray K McKee MD: Treatment of acute midshaft clavicle fractures: Systematic review of 2144 fractures. J Orthop Trauma 2005;19:504-507. 16056089 10.1097/01.bot.0000172287.44278.ef

[37] van der Meijden OA Gaskill TR Millett PJ: Treatment of clavicle fractures: Current concepts review. J Shoulder Elbow Surg 2012;21:423-429. 22063756 10.1016/j.jse.2011.08.053

[38] Frima H van Heijl M Michelitsch C : Clavicle fractures in adults; current concepts. Eur J Trauma Emerg Surg 2020;46:519-529. 30944950 10.1007/s00068-019-01122-4

[39] Wiesel B Nagda S Mehta S Churchill R: Management of midshaft clavicle fractures in adults. J Am Acad Orthop Surg 2018;26:e468-e476. 30180095 10.5435/JAAOS-D-17-00442

[40] Carry PM Koonce R Pan Z Polousky JD: A survey of physician opinion: Adolescent midshaft clavicle fracture treatment preferences among POSNA members. J Pediatr Orthop 2011;31:44-49. 21150731 10.1097/BPO.0b013e3181ff67ce

[41] Bourne M Sinkler MA Murphy PB: Anatomy, Bony Pelvis and Lower Limb, Tibia. Tampa, FL, StatPearls, 2022. 30252309

[42] Lindaman LM: Bone healing in children. Clin Podiatr Med Surg 2001;18:97-108. 11344982

[43] Lindsay SE Alokozai A Eppler SL : Patient preferences for shared decision making: Not all decisions should Be shared. J Am Acad Orthop Surg 2020;28:419-426. 31567900 10.5435/JAAOS-D-19-00146 PMC8080702

[44] Iobst C Arango D Segal D Skaggs DL: National access to care for children with fractures. J Pediatr Orthop 2013;33:587-591. 23812144 10.1097/BPO.0b013e31829b2da4

[45] CMC 2021 Medicare fee-for-service supplemental improper payment data. Available at https://www.cms.gov/httpswwwcmsgovresearch-statistics-data-and-systemsmonitoring-programsmedicare-ffs-compliance/2021-medicare-fee-service-supplemental-improper-payment-data. Accessed July 26, 2022.

